# Upgrading *Pseudomonas* sp. toward Tolerance to a Synthetic Biomass Hydrolysate Enriched with Furfural and 5-Hydroxymethylfurfural

**DOI:** 10.1021/acsomega.4c07288

**Published:** 2025-02-10

**Authors:** Matheus Pedrino, Julia Pereira Narcizo, Inaiá Ramos Aguiar, Valeria Reginatto, María-Eugenia Guazzaroni

**Affiliations:** † Department of Biology, Faculty of Philosophy, Sciences and Letters of Ribeirão Preto, University of São Paulo, Ribeirão Preto, São Paulo 14040-901, Brazil; ‡ Department of Chemistry, Faculty of Philosophy, Sciences and Letters of Ribeirão Preto, University of São Paulo, Ribeirão Preto 14040-900, Brazil

## Abstract

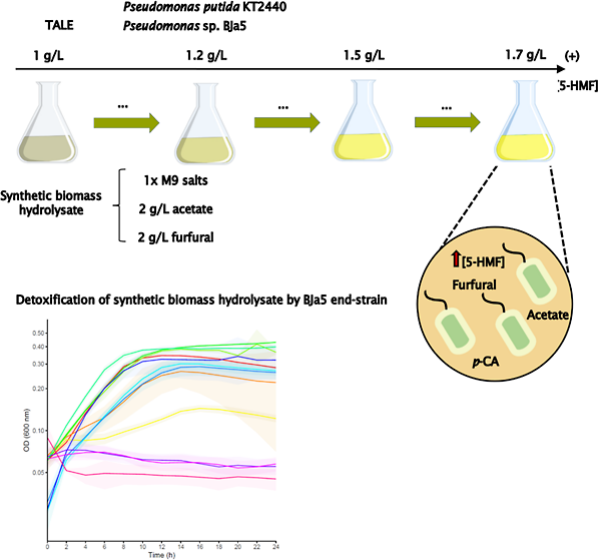

Several *Pseudomonas* species, including *Pseudomonas putida* KT2440, have a broad metabolic repertoire to assimilate biomass monomers such as lignin-derived compounds but struggle to tolerate biomass hydrolysates. Here, we examined the furan derivatives tolerance in a novel and nonpathogenic *Pseudomonas* species (strain BJa5) and in *P. putida* KT2440 using tolerance adaptive laboratory evolution (TALE) to enhance growth performance in a synthetic straw sugar cane hydrolysate enriched with furfural and 5-hydroxymethylfurfural (5-HMF). Initially, wild-type strains showed prolonged lag phases and low tolerance in the synthetic hydrolysate, but tolerance was improved after 90 days of sequential batch growth. Post-TALE, BJa5 and KT2440 end strains grew in synthetic hydrolysate containing 2 g/L furfural and 1 g/L 5-HMF at 48 and 24 h, respectively. Moreover, the KT2440 end strain notably grew in 2 g/L furfural and ≥1.7 g/L 5-HMF. Genome sequencing of end strains revealed mutations in genes and intergenic regions associated with transcriptional factors, acetate metabolism enzymes, environmental response proteins, and transposases. In a proof-of-concept experiment, the BJa5 end strain demonstrated the potential to detoxify synthetic hydrolysate by reducing the titers of acetate and furfural. This ability could enable industrial microorganisms, which are typically nontolerant to toxic hydrolysates, to be used for producing value-added compounds from biodetoxified hydrolysates.

## Introduction

Straw and bagasse sugar cane are common residues from the ethanol industry that have reached from 8 to 15 dry tons per hectare.^1^ In special, straw sugar cane biomass is an abundant raw material composed of cellulose, hemicellulose, and lignin, considered a promising alternative for sugar cane industry and biomass valorization due to potential conversion in high-value bioproducts.^2^ Even available in large quantities, biomass characteristic and recalcitrant lignocellulosic structure of straw sugar cane limit widespread use in industry,^3^ particularly hindering microbial fermentation. The hydrolysates derived from commercial pretreatment methods such as diluted acid and high temperature contain fermentable sugars, weak acids (acetic acid), phenolic compounds, and furfural aldehydes, which are inhibitory for microbial fermentation. To overcome those obstacles, chemical and biotechnological approaches have been employed to reduce biomass recalcitrant effects and ameliorate feedstocks conversion, followed by identification and engineering of new strains that thrive in growth inhibitory environments.^4^

In this context, new microbial nonpathogenic candidates from *Pseudomonas* sp. have been selected for studies using biomass hydrolysates due to inherent tolerance toward aromatic growth inhibitors and a versatile metabolic repertoire, as illustrated by *Pseudomonas putida* KT2440.^5−7^ In special, KT2440 has been recently engineered for catechol and protocatechuic acid biosynthesis using lignocellulosic derived aromatics.^8^ Moreover, an indication that *Pseudomonas* sp. might serve as favorable hosts for biomass valorization is the discovery of new strains such as *P. putida* ALS1267 and *Pseudomonas taiwanensis* VLB120, which harbor genes responsible for degrading furfural and 5-HMF, respectively.^9,10^ Based on that, alternative microbial engineering approaches are becoming essential to explore and establish new *Pseudomonas* sp. strains directed to overcome growth inhibitors stresses.

Lately, microbial engineering tools such as random mutagenesis, genome shuffling, global transcriptional machinery engineering, metabolic engineering, and especially adaptive laboratory evolution (ALE)^11−13^ have been applied to optimize strain tolerance to growth inhibitors and assimilation of lignocellulosic byproducts. However, rational metabolic engineering approaches must go beyond some limitations that reduce the bioprocess yield and increase production costs associated with unstable microbial phenotypes. Rational engineering approaches must be optimized due to phenotypic instability, reduced growth rate, and longer lag phases.^14−16^ Given those limitations, evolutionary approaches such as ALE are promising strategies to develop stable microbial strains in industrial applicability scenarios.^17^ Briefly, ALE is based on continuous culture propagation at exponential phase under selective stress conditions, which may naturally lead to strains with causal mutations and improved fitness. Furthermore, single-point mutations and other genome alterations are identified to comprehend genotype–phenotype causality, enormously facilitated by high-throughput genome sequencing.^18^ For instance, ALE studies have focused on improvements of microbial tolerance to growth inhibitors originated from pretreated biomass hydrolysates^19^ and enriched lignin in *P. putida* KT2440.^20^

In this work, a soil-isolated novel species of *Pseudomonas* (strain BJa5^21^) and *P. putida* KT2440 strain were adapted for 90 days in a synthetic biomass medium enriched in furfural and 5-HMF. The synthetic biomass media formulation was defined according to a straw sugar cane hydrolysate^22^ containing acetate, *p*-coumaric acid (*p*-CA), furfural, and 5-HMF. Whole-genome sequencing was performed to identify the causal mutations acquired along adaptive trajectories in both strains and that might culminate in improved fitness. Moreover, as a proof-of-concept, the potential of the BJa5 end strain to overcome growth inhibitors and reduce the toxicity of synthetic hydrolysate media was assessed. This detoxified medium could subsequently be used by bacteria that are nontolerant to this hydrolysate or to similar toxic biomass hydrolysates resulting from pretreatment. This assessment was conducted using a culture approach with the nonconventional bacterium *Cytobacillus pseudoceanisediminis*, isolated in our laboratory from slurry. The same species had previously been isolated from subsurface saline environments, demonstrating tolerance to heavy-metal-enriched wastes and carrying a set of metal resistance genes.^23^ Thus, the main motivation of this work was to develop new*Pseudomonas* strains that could be used alone or in conjunction with other industrial microorganisms that are naturally nontolerant to toxic hydrolysates. *Pseudomonas* strains generated in this work would serve to detoxify biomass hydrolysates, making them viable as cheap carbon sources for industrial bioprocesses.

## Experimental Section (Materials and Methods)

### Strains and Growth Conditions

*Pseudomonas* sp. BJa5, *P. putida* KT2440, and *C. pseudoceanisediminis* Chr1 strains were cultivated aerobically in a shaker at 200 rpm and 30 °C, using M9 minimal media (47.7 mM Na_2_HPO_4_, 22 mM KH_2_PO_4_, 8.6 mM NaCl, 18.7 mM NH_4_Cl, 2 mM MgSO_4_, 0.1 mM CaCl_2_) or in Lysogeny Broth (LB) media, according to the method employed.

The *C. pseudoceanisediminis* Chr1 was isolated from a sanitary landfill leachate (slurry) concentrated by reverse osmosis in Nova Iguaçu (Rio de Janeiro state, Brazil). The leachate was highly concentrated in metal and refractory organic compounds. Briefly, *C. pseudoceanisediminis* Chr1 was isolated by (i) dilution-plate technique and (ii) directly streaking out the slurry waste in LB agar using an inoculation loop. The plates were incubated at 30 or 37 °C for 24 h, and small colonies were observed for the two techniques. Next, single colonies were carefully picked up and transferred to a new LB agar. After several passage transfers in LB agar, the colony morphology was kept the same. Then, single colonies were inoculated in LB medium to conduct genome sequencing. Genome was sequenced, and based on TYGS genome analysis (Type Strain Genome Server) and digital DNA–DNA hybridization (dDDH), the strain Chr1 was assigned as *C. pseudoceanisediminis* (dDDH4 = 80.9) due to a dDDH4 value > 70%.

For *Pseudomonas* sp. and *Cytobacillus* sp., overnight, preinoculums were, respectively, prepared using M9 media with 2 g/L acetate 1% (v/v) casamino acids and LB media. All chemical reagents and compounds were purchased from Sigma-Aldrich. For endstrain recovery, agar plates containing M9 medium with 2 g/L acetate, 0.3 g/L *p*-CA, 1 g/L furfural, and 0.8 g/L 5-HMF were employed.

### Synthetic Biomass Hydrolysate

The synthetic biomass hydrolysate media was formulated according to growth inhibitors content described in a straw sugar cane hydrolysate pretreated in a microwave associated with diluted sulfuric acid.^22^ Then, the proper formulation of synthetic hydrolysate was experimentally defined using M9 media supplemented with acetate as a carbon source, furfural, *p*-CA, and/or 5-HMF. As total phenolics could not be mimicked, *p*-CA was employed considering that it is the most common lignin-linked phenolic acid present in sugar cane biomass.

The growth tolerance tests in acetate, furfural, *p*-CA, and 5-HMF were performed for BJa5 and KT2440 wild-type clones using at least three biological replicates. Each culture test was initiated at OD_600 nm_ = 0.05 in a 12-well plate (TPP tissue culture plates) incubated at 30 °C and 200 rpm for a proper period (24 or 48 h). The growth was monitored using a VICTOR Nivo Multimode Microplate Reader (PerkinElmer) by end-point measurements at every 2 h. When a 12-well plate was employed in this work, a 3.0 correction factor was applied at OD_600 nm_ measurements to be equivalent to a value measured by a benchtop spectrophotometer using a 1 cm length cuvette. It is noteworthy that 2 g/L acetate was used as the carbon source for furfural, *p*-CA, and 5-HMF growth tests. Each strain was tested using a range concentration for acetate (2.0–6.0–8.0–10–15–20 g/L), furfural (0.5–1.0–2.0 g/L), p-CA (0.016–0.040–0.080 g/L), and 5-HMF (0.5–1 g/L). A stock solution for acetate, *p*-CA, and 5-HMF was prepared in ultrapure water and in alcohol for furfural due to solubility.

### Characterization of Starting Strains

Two versions of synthetic biomass hydrolysate were formulated according to growth screening tolerance in each *Pseudomonas* sp. strain to obtain discrete levels of related stress. The first version was composed of M9 media with 2 g/L acetate, 0.3 g/L *p*-CA, and 2 g/L furfural at pH = 7.0. The second version (harsh stress) was composed of M9 media with 2 g/L acetate, 0.3 g/L *p*-CA, 2 g/L furfural, and 1 g/L 5-HMF at pH = 7.0. The second version was also tested, combining higher acetate contents (5 and 10 g/L) to obtain even harsher stresses. Thus, BJa5 and KT2440 strains were cultivated in both versions of synthetic biomass hydrolysate in 12-well plates at 30 °C and 200 rpm. OD_600 nm_ was monitored in a VICTOR Nivo Multimode Microplate Reader (PerkinElmer) for 24 h to define parameters for tolerance adaptive laboratory evolution (TALE) experiments. The specific growth rates (μ) were calculated for each strain in proper conditions using the linear method.^24,25^

### TALE Experiments

TALE experiments were performed using the continuous batch-culture mode in culture flasks. Single colonies of BJa5 and KT2440 strains were inoculated in M9 media with 2 g/L acetate and 1% (v/v) casamino acids, with at least three biological replicates per strain. Then, the starting cultures (P_0_ #1; P_0_ #2; P_0_ #3) were inoculated in 125 mL flasks containing 10 mL of synthetic biomass hydrolysates (2^nd^ version) at initial OD_600 nm_ = 0.05 with at least three TALE experiments per strain. All flasks were maintained at 30 °C and 200 rpm. OD_600 nm_ was periodically measured in a Biospectrometer (Eppendorf), and at the late-exponential phase, cultures were passaged to fresh synthetic biomass hydrolysate (OD_600 nm_ final = 0.1) to maintain a constant selective pressure. When cultures were adapting during evolution (end point OD_600 nm_ = 0.8–1.0), 5-HMF was gradually incremented from 1 to 1.7 g/L (concentration gradient 1–1.2–1.5–1.7 g/L). TALE experiments were finished at the end of three months when the final OD_600 nm_ per strain was around 0.8 when cultured at 1.7 g/L 5-HMF. Intermediate and end strains were cryopreserved in 20% (v/v) glycerol stocks.

### Characterization of TALE End Strains

BJa5 and KT2440 end strains were recovered from glycerol stocks in M9 agar plates containing 2 g/L acetate, 1 g/L furfural, and 0.8 g/L 5-HMF. Petri dishes were incubated at 30 °C until single colonies were identified (∼2 days). Then, single colonies from parental and end strains were inoculated in M9 medium with 2 g/L acetate and 1% (v/v) casamino acids incubated at 30 °C and 200 rpm overnight. Next, parental and end strains were cultured at initial OD_600 nm_ = 0.05 in a 12-well plate containing 3 mL of synthetic biomass hydrolysate (2 g/L acetate, 2 g/L furfural, and 1 g/L 5-HMF). The 12-well plates were incubated at 30 °C and 200 rpm for at least 24 h, and growth was monitored using a VICTOR Nivo Multimode Microplate Reader (PerkinElmer). Three independent experiments with at least six biological replicates were performed during end strains characterization. The specific growth rates (μ) were calculated for each end strain in proper conditions using the linear method.^24,25^

### Whole-Genome Sequencing

Total genomic DNA of BJa5 and KT2440 parental and end strains were extracted using the GeneJET Genomic Purification Kit (Thermo Fischer Scientific, Waltham, MA, USA) according to the manufacturer’s instructions. The quality and integrity of DNA extracted was evaluated by 1% (w/v) agarose gel electrophoresis, and the concentration of DNA was determined fluorometrically using the Qubit 3.0 (Qubit dsDNA Broad Range Assay Kit, Life Technologies, Carlsbad, CA, USA). The DNA library was prepared using the Nextera XT DNA Library Prep Kit (Illumina, San Diego, CA, USA), assessed for quality using the 2100 Bioanalyzer (Agilent Genomics, Santa Clara, CA, USA), and subsequently submitted to sequencing using the Illumina HiSeq (2 × 150 bp) platform (Illumina, San Diego, CA, USA). Raw data was preprocessed using the fastp tool (v.0.23.4) to remove low-quality sequences. Only sequences with quality > Q20 were selected to the assembly step that was executed using the software Unicycler (v.0.5.0). Next, the assembled genomes were submitted to functional annotation using Prokka (v.1.14.6), followed by functional mapping with eggNOG-mapper (v.2.1.12). To detect fixed mutations after TALE, BJa5, and KT2440 end-strains genomes were compared with reference genomes (BJa5 GenBank accession number JALRNG000000000.1 and KT2440 GenBank accession number JBFMZO000000000) using ParSNP (v.1.7.4) and NucDiff (v2.0) focusing on single nucleotide polymorphisms (SNPs). Then, the mutated regions were mapped into annotation files to precisely identify the genomic alterations.

### Detoxification of Synthetic Hydrolysate by BJa5 End Strains

The BJa5 end strains were precultured overnight and inoculated in 15 mL of synthetic hydrolysate (2 g/L acetate, 2 mM *p*-CA, 2 g/L furfural, and 1 g/L 5-HMF) using 125 mL culture flasks. After 48 h, the cells were harvested at 10.000 rpm for 10 min, and the supernatant was collected and filtered using a 0.22 μm syringe filter (Biofil). Next, *C. pseudoceanisediminis* Chr1 isolate was cultivated in detoxified and undetoxified (filtered supernatant) synthetic hydrolysates at 30 °C and 200 rpm for 24 h. OD_600 nm_ was monitored using a VICTOR Nivo Multimode Microplate Reader (PerkinElmer) by end-point measurements at every 2 h. At least six biological replicates were employed in this assay.

### HPLC Analysis

Acetate, furfural, and 5-HMF were analyzed on a high-performance liquid chromatogram (Shimadzu LC-20 A T, Japan) equipped with an Aminex HPX-87H column. The column temperature was maintained at 60 °C; the mobile phase was 5 mmol/L H_2_SO_4_ at 0.6 mL min (84 Kgf. cm^2^). A RID detector was employed, and the data was processed with the software LabSolutions. An analytical curve of 99% (v/v) furfural and 5-HMF (Sigma, USA), from 0.01 to 8 g/L, aided furfural, 5-HMF, and acetic acid quantification.

### Statistical Analysis

R version 4.4.0 was employed for all statistical analyses performed in this work. One-way analysis of variance and Tukey post hoc were used to compare OD_600 nm_ measurements and growth rate data among groups. The significance level was adopted as 5% with *p* *< 0.05, *p* **< 0.01, and *p* *** < 0.001. The Tidyverse packages (v. 1.3.2) were used for visualizing data and graphs.

## Results and Discussion

### Formulating a Synthetic Hydrolysate Medium

**Figure 1:**
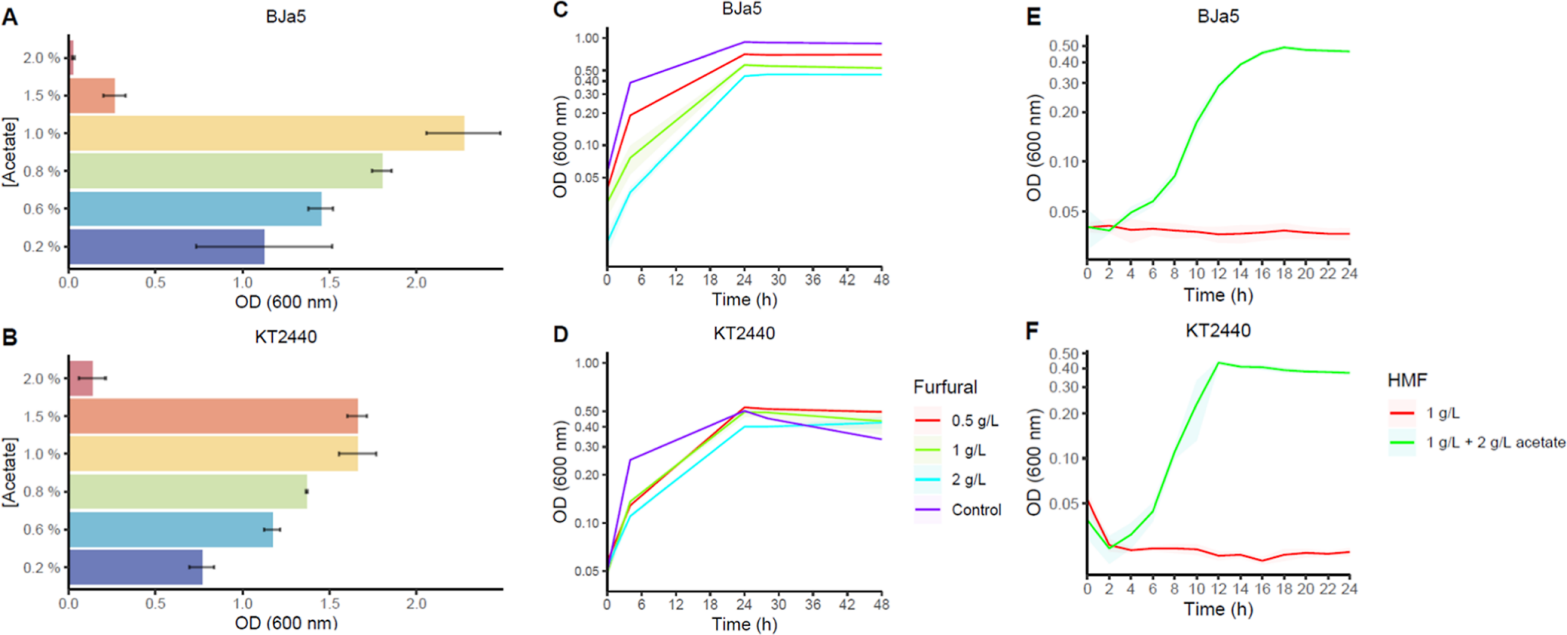
Formulating a synthetic biomass hydrolysate. End-point OD_600 nm_ from (A) *Pseudomonas* sp. BJa5 and (B) *P. putida* KT2440 cultured in acetate gradient (0.2, 0.6, 0.8, 1, 1.5, and 2%*) at 24 h. *Pseudomonas* sp. BJa5 (C) and *P. putida* KT2440 (D) growth curves in M9 media+ 2 g/L acetate with 0.5, 1, and 2 g/L furfural. The control group was grown in M9 media+ 2 g/L acetate. *Pseudomonas* sp. BJa5 (E) and *P. putida* KT2440 (F) cultured in M9 media+ 2 g/L acetate +1 g/L 5-HMF and in M9 media solely with 1 g/L 5-HMF. The plots represent mean + SD (smooth lines) from at least three biological replicates. *(%) units correspond to 2, 6, 8, 10, and 20 g/L. A 3.0 factor should be applied to correct optical path lengths for panels C, D, E, and F.

Based on a straw sugar cane hydrolysate described by Fonseca and colleagues, BJa5 and KT2440 strains were cultivated in 2 g/L acetate as the carbon source combined with *p*-CA, furfural, or 5-HMF to formulate a proper synthetic hydrolysate media (Figure 1). The *P. putida* KT2440 strain was more tolerant than BJa5 cultured only in acetate, being capable of growing at 15 g/L acetate in 24 h (Figure 1B). Both strains had growth inhibitory effects at 20 g/L acetate (Figure 1A,B). It was reported that *P. putida* KT2440 can efficiently grow in 10 g/L acetate.^26^ Interestingly, the *Pseudomonas* sp. BJa5 strain could achieve higher OD_600 nm_ levels than KT2440 when cultured at 10 g/L acetate (1%), this being a potential microbial platform to be applied in biomass feedstocks or similar wastes. Moreover, acetate is a compound highly enriched in sugar cane hydrolysates, attaining between 3.5 and 10 g/L,^27,28^ a suitable range for growing of both strains. In addition, microbial acetate conversion in tricarboxylic acid and glyoxylate intermediates could be explored for bioproducts, especially organic acids.^29^ Then, aligned with the acetate content and tolerance results for both strains (Figure 1A,B), 2 g/L acetate was selected as the suitable concentration for synthetic biomass media.

Next, M9 media + 2 g/L acetate was combined with *p*-CA (0.016–0.040–0.080 g/L), which did not promote any inhibitory effect for both strains demonstrated by the short lag-phase and fast growing within 4 to 6 h (Supporting Information: Figure S1. Growth characterization of starting strains in the *p*-CA gradient). The rapid growth observed in KT2440 may be explained by the combined use of acetate and *p*-CA as carbon sources, as well as degradation pathways (*fcs*, *ech*, and *vdh* genes) that are linked to tolerance mechanisms for maintaining membrane integrity.^30^

Concerning furan aldehydes, BJa5 and KT2440 strains were cultivated on M9 media + 2 g/L acetate with furfural (0.5, 1, and 2 g/L) for 48 h and 1 g/L 5-HMF for 24 h. Neither furfural nor 5-HMF affected growth of both strains when they were associated with 2 g/L acetate (Figure 1C–F) which surpassed the inhibitory effects of each furan aldehyde. As expected, wild-type strains were not capable of growing only with 5-HMF as the carbon source (Figure 1E,F). Furfural and 5-HMF metabolic pathways are described in several bacteria like*P. putida* strains Fu1 and F2,^31,32^*P. putida* ALS1267,^9^ and *Cupriavidus basilensis* HMF14.^33^ Moreover, some *Pseudomonas* species, particularly *P. putida* KT2440,^5^ have been reported to tolerate aldehydes. This tolerance could provide insights into unknown mechanisms and reactions for transforming furan aldehydes into less toxic compounds. Recently, primary HMF-converting enzymes were identified in a *P. taiwanensis* VLB120 isolate and in *P. putida* KT2440 due to new oxidoreductase and dehydrogenase activities.^10^ Similarly, the BJa5 strain showed results comparable to those of KT2440 (Figure 1), prompting its application in synthetic media characterization and TALE experiments in this work.

### Starting Strains Characterization in Synthetic Biomass Media

**Figure 2:**
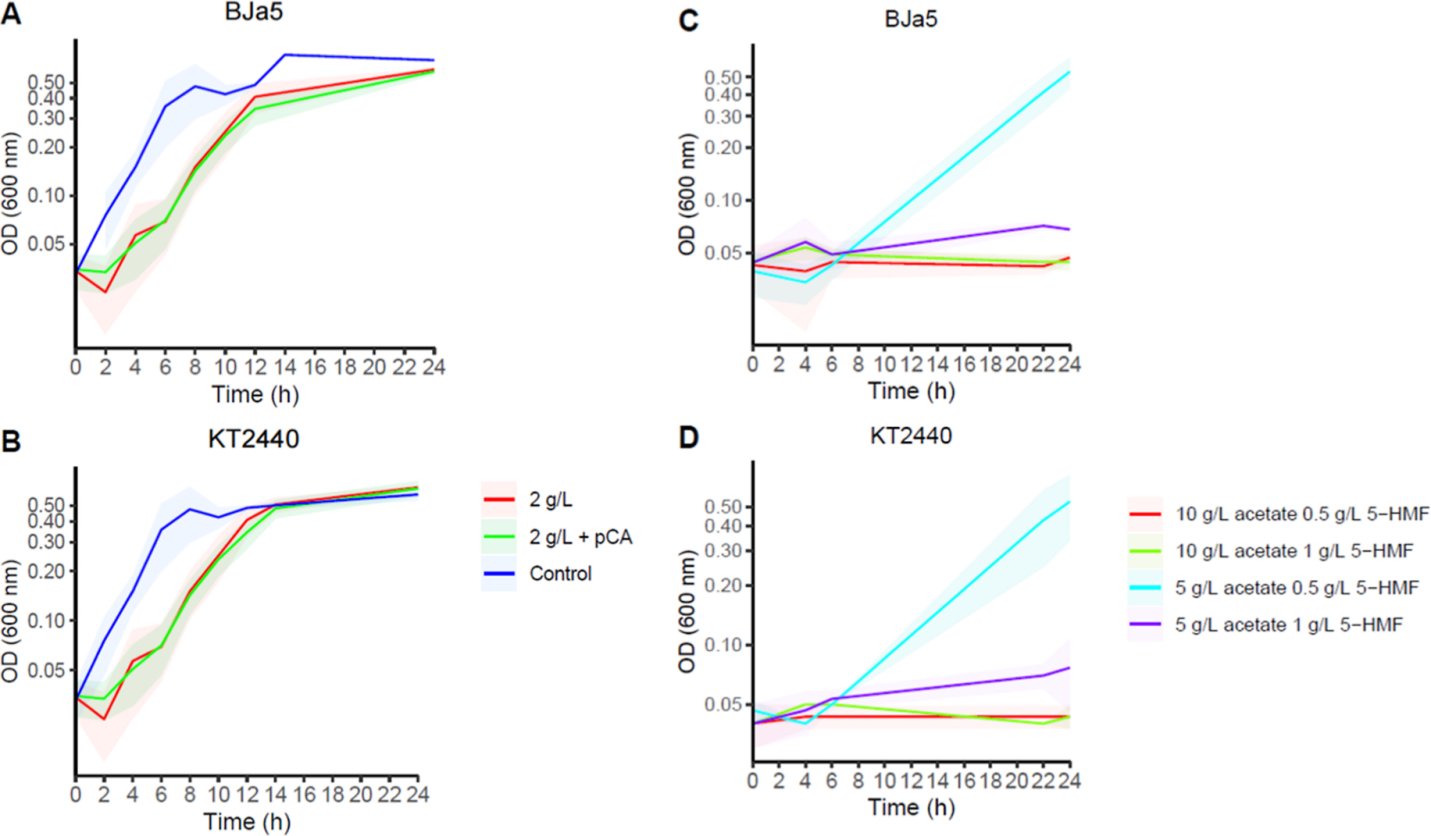
Characterization of starting strains using batch culture in synthetic biomass hydrolysate. (A) *Pseudomonas* sp. BJa5 and (B) *P. putida* KT2440 cultivated in a 1^st^ version of synthetic biomass hydrolysate (2 g/L acetate, 0.3 g/L *p*-CA, 2 g/L furfural) at 24 h. The control group (blue line) was represented by BJa5 and KT2440 growing in M9 media only with 2 g/L acetate. *Pseudomonas* sp. BJa5 (C) and *P. putida* KT2440 (D) cultivated in a 2^nd^ version of synthetic biomass hydrolysate containing 5 or 10 g/L acetate, 0.3 g/L *p*-CA, 2 g/L furfural, and 0.5 or 1 g/L 5-HMF. A 3.0 factor should be applied to correct optical path lengths for panels A, B, C, and D.

To investigate the combined effect of furfural and 5-HMF, BJa5 and KT2440 strains were cultured in synthetic biomass media containing acetate, *p*-CA, and furfural and in the same media with addition of 5-HMF for 24 h. Similar growth profiles were observed for both strains cultured in the first version of synthetic hydrolysates (M9 media with 2 g/L acetate, 0.3 g/L *p*-CA, 2 g/L furfural) showing prolonged lag phases within 8 h compared to the control group (only fed with 2 g/L acetate) (Figure 2A,B). Moreover, BJa5 and KT2440 strains achieved similar maximum OD_600 nm_ values compared to the control group (0.5–0.6). No significant differences were observed between the groups fed only with furfural and those fed with furfural + *p*-CA. This is likely because *p*-CA is rapidly assimilated under aerobic conditions due to its simpler metabolism, unlike in anaerobic cultures of *Clostridium* sp.,^34^ explaining the lack of differences between the groups. On the other hand, *p*-CA alone might not replicate the same inhibitory effects observed in raw hydrolysates due to the complexity of the phenolics fraction. However, differences in growth rates were observed among strains grown in the first version of synthetic hydrolysate (0.23–0.25 h^–1^) compared to the group fed only with 2 g/L acetate (0.35 h^–1^).

5-HMF is a degradation product commonly found in liquid pretreatment fractions^35^ and is easily obtained from sugar cane hydrolysates. As expected, when 5-HMF and furfural were combined, higher growth inhibitory effects were observed in BJa5 and KT2440 strains compared with the later version of the synthetic media. Unlike furfural, 5-HMF is considered less inhibitory to KT2440 and is assimilated after furfural removal.^36^ Nevertheless, several inhibitory compounds together can elicit synergistic effects, as observed in biomass hydrolysates.^35^ Our results show that increased acetate titers in association with 1 g/L 5-HMF promoted more toxic effects in both strains than previously observed (Figure 2C,D). After 3–4 days, no increase in OD_600 nm_ was observed for BJa5 and KT2440 when cultivated in synthetic biomass with 1 g/L 5-HMF. Similar results were observed when the synthetic hydrolysate was composed of 2 g/L acetate and 1 g/L 5-HMF, reinforcing the coupled inhibitory potential of furan aldehydes (data not shown). Additionally, BJa5 and KT2440 were reproducibly cultured after a long lag phase (Figure 2C,D) in synthetic biomass media, making them suitable to start TALE experiments.

### Characterization of End Strains (TALE)

For TALE experiments, at least three clones of BJa5 and KT2440 strains were cultured in synthetic hydrolysate media containing M9 media with 2 g/L acetate, 2 g/L furfural, and a 5-HMF gradient (1, 1.2, 1.4, and 1.7 g/L). During TALE trajectories, differences in final OD_600 nm_ per passage were crucial for increasing 5-HMF concentrations and maintaining constant selective pressure (5-HMF gradient from 1 to 1.7 g/L was used). In the last days of evolution, both strains serially cultured in 1.7 g/L of 5-HMF exhibited shorter lag phases (<20 h) and enhanced OD_600 nm_ (0.6–0.8). Potentially evolved strains were recovered and compared with wild-type growth performances. Characterization of the end strains revealed BJa5 and KT2440 clones reproduced the growth improvements observed during TALE experiments when they were cultured in synthetic biomass media enriched with furan aldehydes.

**Figure 3:**
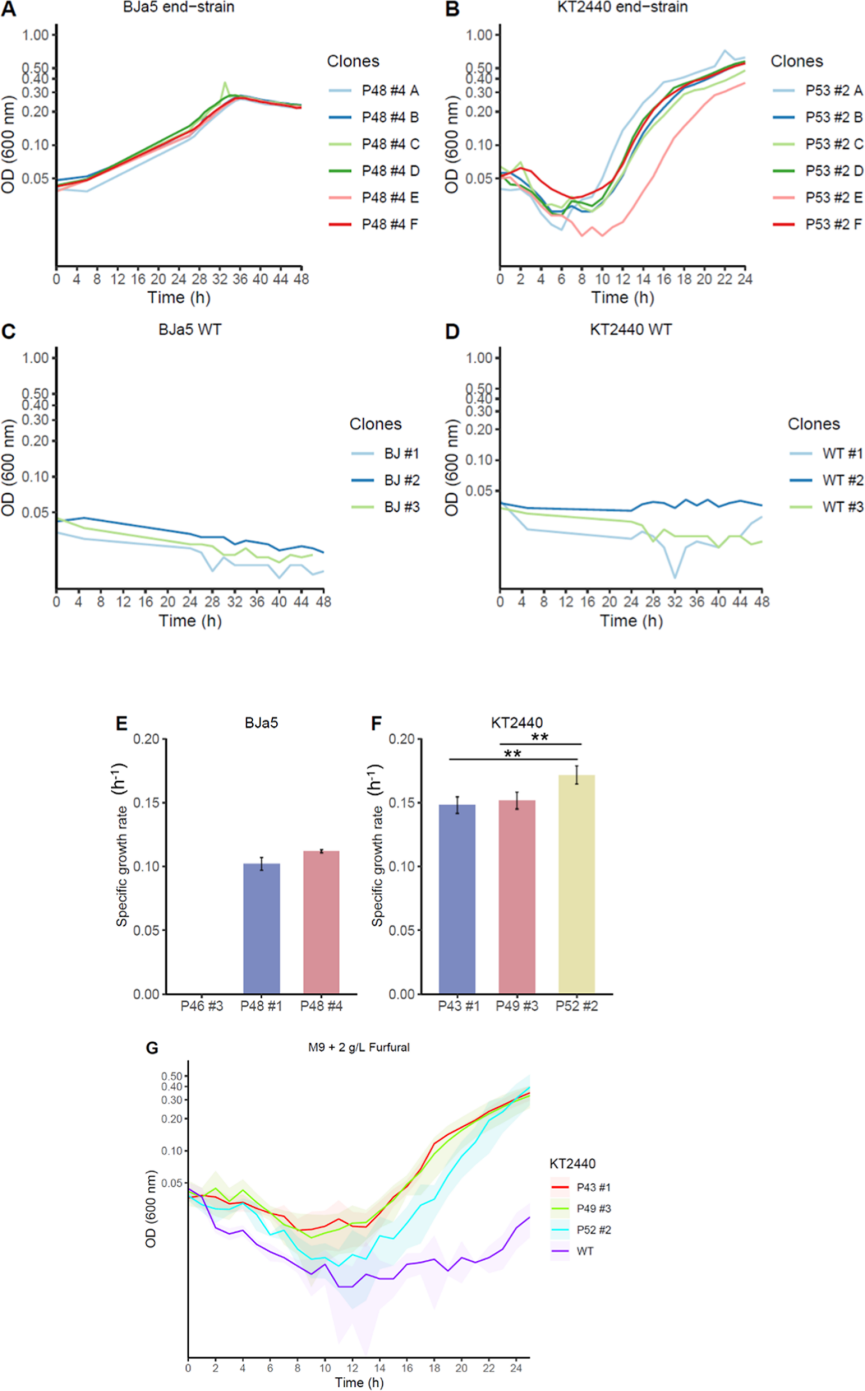
Growth characterization of BJa5 and KT2440 TALE-derived strains. *Pseudomonas* sp. BJa5 P48 #4 (A) and *P. putida* KT2440 P53 #3 (B) and, respectively, wild-type clones (C,D) cultivated in synthetic biomass hydrolysate (2 g/L acetate, 0.3 g/L *p*-CA, 2 g/L furfural, 1 g/L 5-HMF). Evolved strains were recovered after 3 months of continuous batch growth in 125 mL flasks using a 5-HMF gradient from 1 to 1.7 g/L. The bar plot shows the specific growth rate (μ ± SD) calculated for BJa5 (E) and KT2440 (F) end strains considering at least three technical replicates in synthetic biomass hydrolysate (2 g/L acetate, 0.3 g/L *p*-CA, 2 g/L furfural, 1 g/L 5-HMF). (**) represents *p* < 0.01. (G) *P. putida* KT2440 end strains cultivated in M9 with 2 g/L furfural as the sole carbon source for 24 h. A 3.0 factor should be applied to correct optical path lengths for panels A, B, C, D, and G.

Among the TALE replicates studied, BJa5 from passage 48 replicate 4 (P48 n^o^. 4) and KT2440 from passage 53 replicate 2 (P53 n^o^. 2) exhibited the shortest lag phase and highest growth rate. The end strains BJa5 P48 #4 and KT2440 P53 #2 were able to exclusively grow in synthetic hydrolysate containing 1 g/L 5-HMF within 48 and 24 h, respectively (Figure 3A,B). Unlike BJa5 end strains, KT2440 P53 #2 and KT2440 P49 #3 end strains could be cultivated in synthetic hydrolysate containing up to 1.7 g/L, achieving end-point OD_600 nm_ values between 3.4 and 4.2 in 12-well plates within 48 h. Regarding the growth rate, all KT2440 end strains achieved higher growth levels compared to both BJa5 and wild-type strains (Figure 3E,F). Despite these differences, BJa5 P48 #1 and #4 replicates consistently showed growth in synthetic biomass media across three independent experiments, underscoring the potential of these new BJa5 end strains.

Notably, KT2440 end strains exhibited rapid growth after reaching the exponential phase, suggesting that inhibitory effects induced by furfurals were overcome, possibly through conversion to less-toxic compounds or assimilation. This result motivated an investigation into the capability of KT2440 end strains to utilize furfural as a sole carbon source, a trait probably acquired during the TALE experiments. Remarkably, all KT2440 end strains (P43 #1, P52 #2, and P49 #3) were able to grow in M9 medium supplemented with 2 g/L furfural within 25 h, demonstrating promising resilience against the toxicity of raw biomass hydrolysates (Figure 3G). This finding elucidates the significant growth enhancements observed in KT2440 end strains when cultivated in synthetic biomass hydrolysates containing up to 1.7 g/L 5-HMF, underscoring the importance of furfural conversion in mitigating media toxicity. Moreover, previous ALE experiments with *P. putida* exposed to furfural and 5-HMF have reported that reduced furfural levels enable assimilation of available carbon sources into the central metabolism,^36^ which corroborates these findings. In contrast, BJa5 end strains did not exhibit any increase in OD_600 nm_ when furfural was provided as the sole carbon source (data not shown).

Generally, only a few microorganisms possess the ability to degrade furfurals, such as certain aerobic Gram-negative bacteria from genera like *Cupriavidus* sp., *Burkholderia* sp., and *Methylobacterium* sp.^37^ Additionally, there are numerous reports regarding species within the *Pseudomonas* genus, exemplified by certain *P. putida* strains capable of metabolizing furanic compounds, such as 2-furoic acid and furfural. For instance, evolved strains of *P. putida* ZL efficiently converted 1 g/L of furfural and 1 g/L of 5-HMF within 24 h in simulated hydrolysates containing 5 g/L of acetate.^36,38^ In another study, evolved strains of *Pseudomonas pseudoalcaligenes* CECT 5344 demonstrated the ability to utilize furfural (≥40 mM), 2-furoic acid (≥20 mM), and furfural alcohol (≥20 mM).^39^ This study has contributed to the development of new strains within the *Pseudomonas* genus, particularly KT2440 end strains, which exhibit tolerance to furfural (2 g/L) and 5-HMF (1.7 g/L). These strains could potentially serve as microbial platforms for the bioremediation of biomass hydrolysates.

### Potential Key Mutations Identified in End Strains

**Figure 4:**
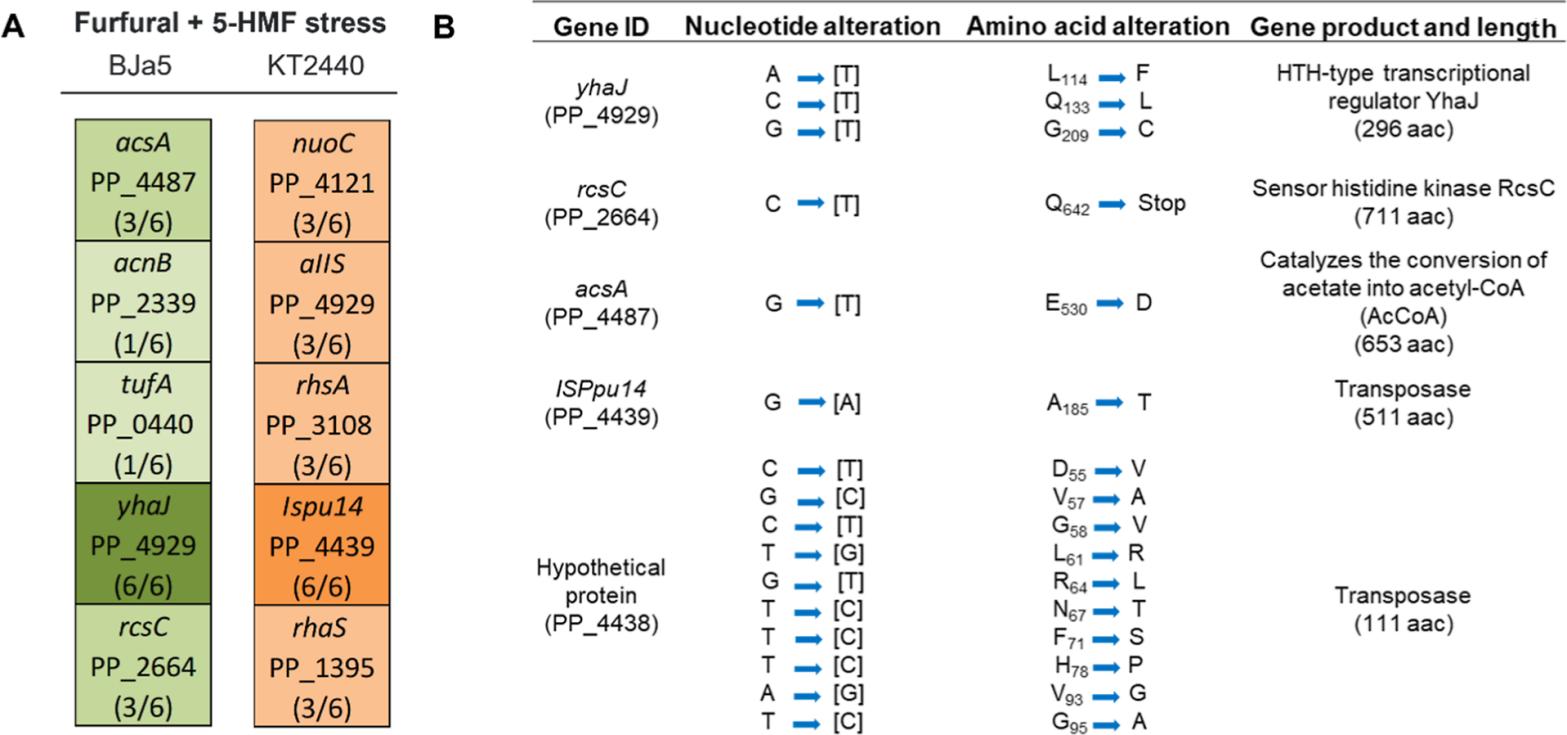
Most mutated genomic regions in BJa5 and KT2440 end strains (six biological replicates) under furfural and 5-HMF stress. (A) The numbers in parentheses indicate the number of TALE replicates in which mutations in the specified gene were observed in at least one strain. Gene annotation was based on the GenomeScan file for each strain. (B) Nonsynonymous mutations of the main mutated genes in BJa5 end strains (*yhaJ*, *rcsC*, and *ascA*) and in KT2440 end strains (*ISPpu14* and hypothetical protein). Each number subscribed represents the amino acid position changed.

To gain deeper insights into the growth enhancements observed following TALE experiments, detailed genomic analyses were conducted on the BJa5 and KT2440 end strains to identify mutations (SNPs) compared to their respective wild-type reference genomes. Six biological replicates per end strain were sequenced (Figure 4). The numbers in parentheses shown in Figure 4 indicate the number of TALE replicates in which mutations in the specified gene were observed in at least one strain. In BJa5 end strains, 23 mutations were identified, affecting 6 annotated genes, 3 genes encoding hypothetical proteins, and 14 intergenic regions across the genomes. Similarly, KT2440 end strains exhibited 16 mutations, impacting 7 annotated genes, 3 genes encoding hypothetical proteins, and 6 intergenic regions.

The most mutated genomic regions in BJa5 end strains were identified in genes involved in transcriptional regulation (*yhaJ* PP_4929), mutated in all 6 replicates; in the conversion of acetate to acetyl-CoA (*acsA* PP_4487), mutated in 3 out of 6 replicates; and in response to environmental cues (*rcsC* PP_2664), also mutated in 3 out of 6 replicates. In KT2440 end strains, the most mutated genomic regions were found in genes encoding ISPpu14 transposase (PP_4439), which was mutated in all sequenced end strains (Figure 4A). Interestingly, all identified SNPs in BJa5 end strains were nonsynonymous mutations, whereas approximately 37.5% (12 out of 32 mutations) of SNPs in KT2440 end strains were in transposase genes and were nonsynonymous (Figure 4B). However, further investigation is required to determine whether these changes significantly affect the structure, stability, and function of the resulting proteins.

Notably, mutations were identified in HTH-type regulator genes encoding transcriptional activators YhaJ (LysR family) and RhaS (AraC type) in BJa5 and KT2440 end strains, respectively (Figure 4A). These mutations likely impact the function of the *yhaJ* and *rhaS* genes (this late, also mutated in KT2440), potentially altering the expression of multiple genes that might play a role in environmental adaptation. This scenario is reminiscent of observations in *Escherichia coli* with the degradation of aromatic compounds like 2,4-dinitrotoluene (DNT) by a LysR family protein.^40^ Moreover, AraC-type regulators participate in numerous cellular processes, especially those associated with carbon metabolism^41^ and stress response^42^ in bacteria.

In particular, YhaJ may play a crucial role in cellular responses to toxic compounds such as furfural, facilitating adaptation and bacterial survival in challenging environments. The LysR family encompasses a diverse array of transcriptional regulators with roles in various cellular processes, including carbon source assimilation, which could influence the utilization of furfural as a carbon source.^43^ In a related context was reported a mutated *araC* family gene (also a regulator of carbon source assimilation) enabling *P. pseudoalcaligenes* to utilize furfural as a carbon source.^39^ This suggests that the metabolism of furfural in BJa5 end strains may be linked to the mutated *yhaJ* gene, despite its inability to grow on furfural as the sole carbon source.

Furthermore, in BJa5 end strains, a mutation in the *rcsC* gene introduced a premature termination codon, which should affect the protein’s structure. RcsC regulates stress response pathways, including capsule synthesis, motility, and biofilm formation.^44^ ALE studies frequently identify mutations in flagellar genes and other motility-related components, which reduce energy consumption.^20,45^ Also, this disruption in the *rcsC* gene might be associated with energy consumption maintenance in BJa5 end strains, which must be further investigated.

**Figure 5:**
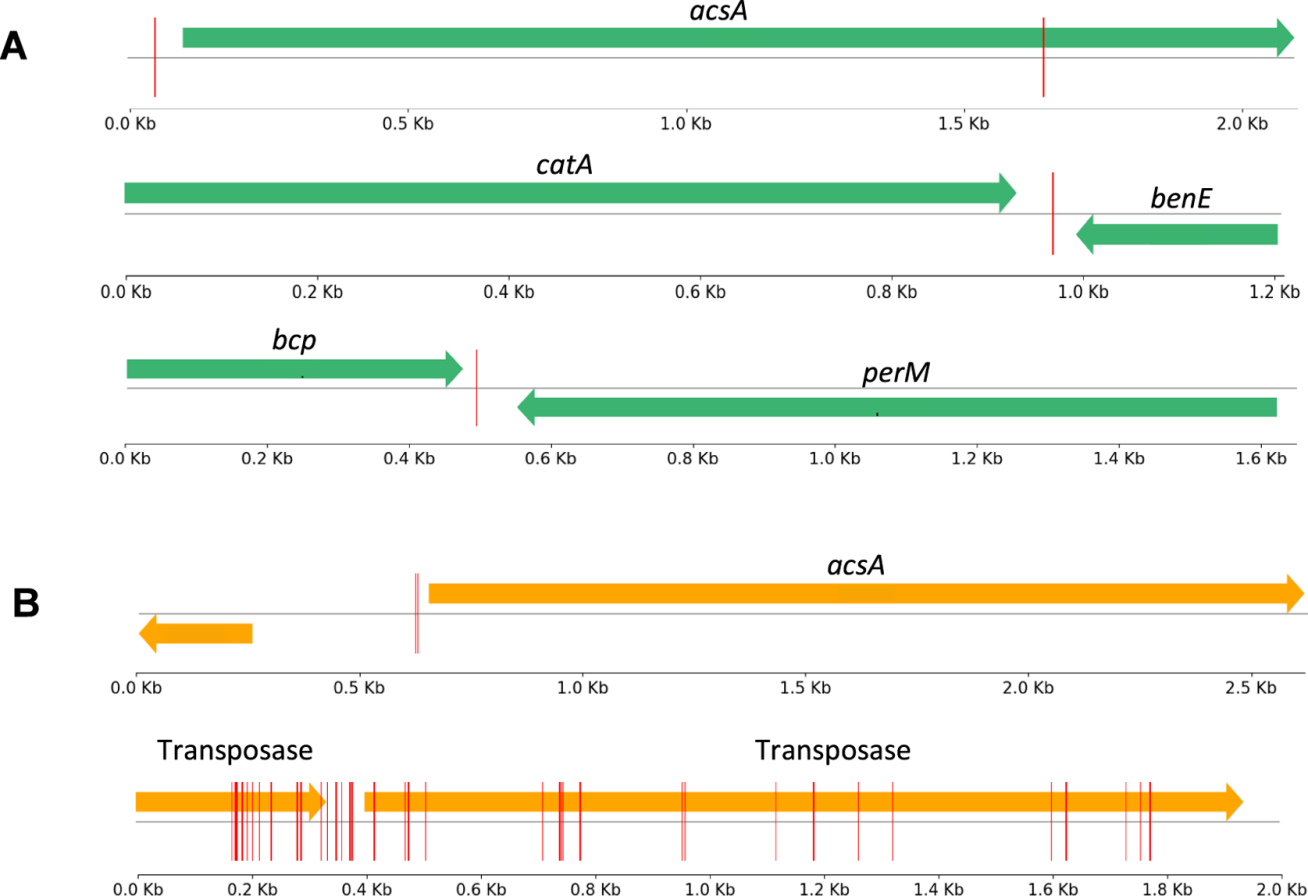
Graphic representation of most mutated intergenic regions in *Pseudomonas* sp. BJa5 (A) and *P. putida* KT2440 (B) end strains under furfural and 5-HMF stress. The red vertical lines represent the position identified for each SNP.

Numerous identical mutations were identified in all KT2440 end-strain replicates located in genes coding for transposases IS66 and in the respective intergenic region (Figure 5B). Unlike *Pseudomonas* sp. BJa5 that contains two IS3 family transposases, *P. putida* KT2440 has 36 insertion elements (IS) that might be associated with resistance obtained by DNA rearrangements or horizontal gene transfer,^46^ affecting the expression of acquired functions and variability. Moreover, the transposases genes mutated were located next to genes with interesting functions, like a zinc-dependent alcohol dehydrogenase (YbdR PP_3970), which has been previously documented as playing a role in the oxidation of ethylene glycol to glyoxylate in *P. putida* KT2440^47^ and associated with furfural detoxification in microbes.^48^ Also, in KT2440 end strains were identified mutations in the *rhsA* gene PP_3108 (Figure 4A), encoding a YD-peptide repeat protein which is described as a rearrangement hotspot region being a site for recombination in *E. coli*(49) and sharing some functions related to growth inhibition and cell wall interaction.^50^

As anticipated, both end strains exhibited mutations in intergenic regions upstream of the *acsA* gene, which encodes an acetyl-CoA synthetase A responsible for activating acetate in microbial metabolism (Figure 5A,B). Intergenic mutations can influence gene regulation and expression of neighboring genes. In this context, alterations in the regulation of *acsA* could potentially enhance acetate assimilation efficiency, thereby providing the necessary energy and precursors for survival in challenging environments containing inhibitory compounds.

Additionally, another intergenic mutation was identified upstream across the genome, with BJa5 showing a mutation near the metal-pseudopaline receptor CntO in 4 out of 6 replicates. Metal pseudopalines are molecules capable of chelating essential metals like zinc, nickel, and particularly cobalt, crucial for *Pseudomonas aeruginosa* in biofilm production under low-oxygen conditions.^51−53^ The *cntO* gene (PP_3325) or ferric siderophore receptor encodes an outer membrane component involved in pseudopaline recovery from the external environment.^54^ These discoveries provide valuable insights for future investigations into the molecular mechanisms driving the adaptation of *Pseudomonas* sp. in challenging environments.

### Detoxification of Synthetic Biomass Hydrolysates by the *Pseudomonas* sp. BJa5 End Strain

Given that *P. putida* KT2440 is a well-established microbial host, our focus shifted to exploring new insights into the BJa5 end strain, particularly its potential for detoxifying synthetic hydrolysates, likely through the conversion of furfurals into less toxic compounds.^37,55^ As evidenced, the BJa5 end strain exhibits the capability to thrive in synthetic biomass hydrolysates enriched with furan aldehydes, notably, 5-HMF and furfural. This marks the first report on the *Pseudomonas* sp. BJa5 strain showcases promising traits applicable in furfural-enriched media, potentially including biomass hydrolysates.

Building on this, nine clones derived from the BJa5 P48 #1 end strain were cultivated in synthetic hydrolysate (2 g/L acetate, 2 mM p-CA, 2 g/L furfural, 1 g/L 5-HMF) for 48 h. Cell-free supernatants, termed detoxified synthetic media, were collected. Then, a furfural-susceptible *C. pseudoceanisediminis* bacterium, isolated in our laboratory from concentrated slurry, a liquid waste, was cultivated in both detoxified and undetoxified synthetic media versions for 24 h as a proof-of-concept.

**Figure 6:**
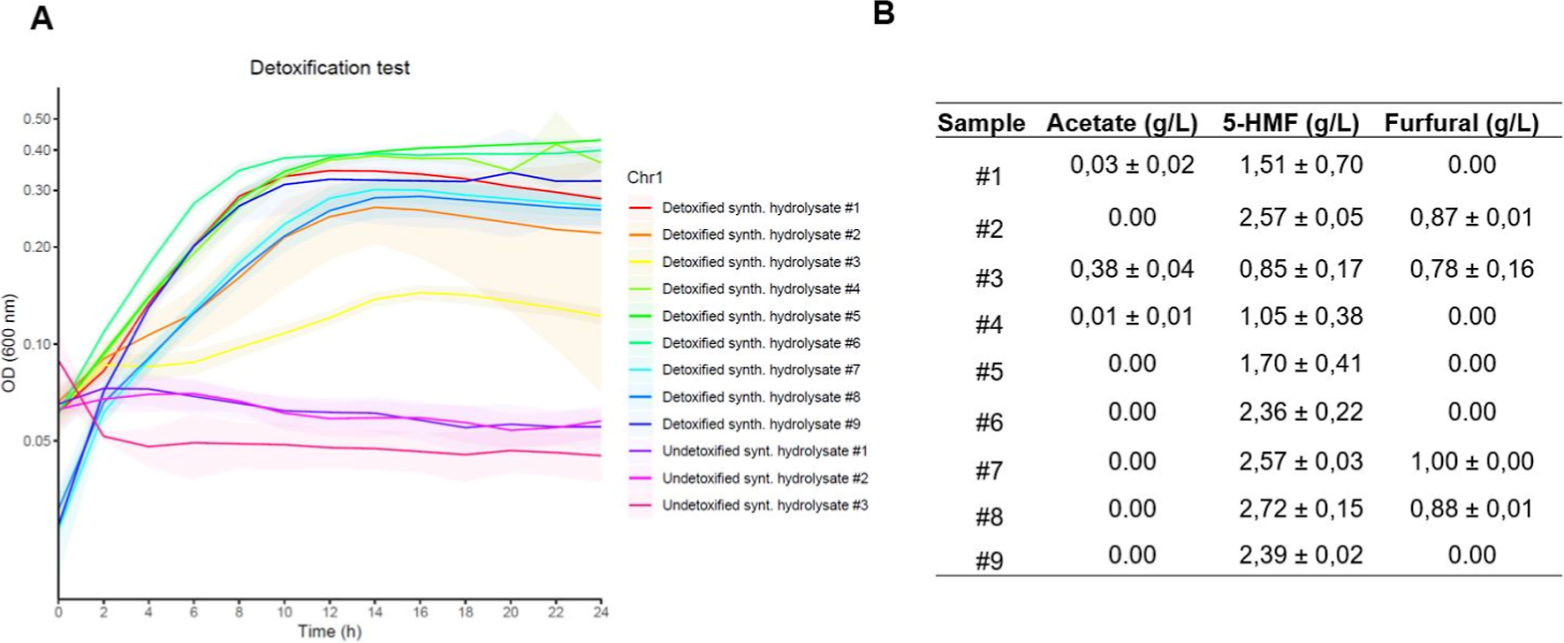
Detoxification potential of the BJa5 end strain. (A) Growth of *C. pseudoceanisediminis* Chr1 in synthetic hydrolysate detoxified by the BJa5 end strain. The plots represent mean + SD (smooth lines) from at least three biological replicates. A 3.0 factor should be applied to correct optical path lengths for panel A. (B) Acetate, 5-HMF, and furfural content in synthetic hydrolysates detoxified by BJa5 end-strain clones determined by HPLC analysis. The synthetic media for detoxification assay contained 1.6–2.0 g/L acetate, 1.7–2.0 g/L furfural, and 1.7–2.0 g/L 5-HMF.

Remarkably, *C. pseudoceanisediminis* reached rapid growth and a higher maximum OD_600 nm_ around 0.4–0.45 for at least four clones tested in detoxified media, but growth was hampered in undetoxified synthetic media (Figure 6A). This result corroborated the growth performance of *Pseudomonas* sp. BJa5 is observed in Figure 3A, demonstrating that the BJa5 P48 #1 clone has potential to detoxify synthetic hydrolysate enriched with furfural.

In parallel, HPLC analysis (Figure 6B) showed that almost all detoxified media collected had reduced furfural content to low (0.8 g/L) or undetectable levels, especially for clones #1, #6, and #9, proving that the BJa5 end strain could assimilate furfural at 2 g/L. Additionally, acetate levels in detoxified media were diminished or undetectable, indicating that acetate was assimilated as a carbon source by the end strains. Noteworthily, when acetate, furfural, and 5-HMF were present in detoxified media, higher inhibitory effects were observed in *C. pseudoceanisediminis* as shown in sample #3. This supports the hypothesis that growth inhibitors in synthetic hydrolysate might be acting in combination. Furthermore, 5-HMF was selectively consumed with high titers present in some detoxified media samples, reinforcing the hypothesis that furfural toxicity is a primary challenge that must be overcome by bacteria.

Similar results were observed with a detoxifying KT2440 strain engineered to consume growth inhibitors in a microbial consortium with *Bacillus coagulans*.^38^ Generally, few natural microbes can tolerate or detoxify inhibitory compounds while consuming fermentative sugars available in biomass hydrolysates.^56^ Therefore, generation of strains with detoxification abilities, as demonstrated by *Pseudomonas* sp. BJa5 end strain could significantly enhance biomass valorization. This approach would allow conventional industrial microorganisms naturally nontolerant to inhibitory compounds present in toxic hydrolysates to be applied to these detoxified hydrolysates to produce value-added compounds.

Given that the BJa5 end strain has the potential to reduce the inhibitory effects of synthetic hydrolysate, future efforts should be directed toward understanding how growth performance is affected in raw biomass hydrolysates potentially enriched with furfurals. Despite some limitations in accurately quantifying 5-HMF and replicating phenolic fraction effects with *p*-CA, formulation of the synthetic hydrolysate proposed in this work was reproducible and effective in maintaining stress-selective pressure in TALE experiments, as observed by the potential of the end strains.

Additionally, the *P. putida* KT2440 end strain showed better growth traits than did the*Pseudomonas* sp. BJa5 end strain, possibly due to the usage of furfural as a carbon source. This fitness improvement should be further explored in raw hydrolysates. Finally, SNPs identified in both end strains supported the findings after TALE characterization, showing alterations in genes related to acetate metabolism, transcriptional regulators, and sensor proteins in the end strains.

## Data Availability

*Pseudomonas* sp. BJa5 and *P. putida* KT2440 genome sequences data were deposited in the NCBI GenBank database, respectively, under accession numbers JALRNG000000000.1 and JBFMZO000000000.

## Supporting Information Available

Summary of the strains used in this work along with the set of mutations identified in tolerant BJa5 and KT2440 and information on the growth characterization of BJa5 and KT2440 starting strains in M9 media+ 2 g/L acetate + p-CA (0.016–0.040–0.080 g/L), which did not promote any inhibitory effect for both strains (PDF)
