# Supplementary material for: Upgrading Pseudomonas sp. toward Tolerance to a Synthetic Biomass Hydrolysate Enriched with Furfural and 5-Hydroxymethylfurfural
Source: ACS Omega. 2025 Feb 10;10(6):5449–59. doi: 10.1021/acsomega.4c07288 (PMC11840610; doi:10.1021/acsomega.4c07288)
Supplement: Supplementary file 1 — ao4c07288_si_001.pdf [file ao4c07288_si_001.pdf]

Upgrading *Pseudomonas* sp. towards tolerance to a synthetic biomass hydrolysate enriched with furfural and 5-hydroxymethylfurfural

Matheus Pedrino[1], Julia Pereira Narcizo[2], Inaiá Ramos Aguiar[1], Valeria Reginatto[2], and María-Eugenia Guazzaroni[1*]

[1]Department of Biology, Faculty of Philosophy, Sciences and Letters of Ribeirão Preto, University of São Paulo, São Paulo, SP, Brazil

[2]Department of Chemistry, Faculty of Philosophy, Sciences and Letters of Ribeirão Preto, University of São Paulo, Ribeirão Preto, Brazil

*Correspondence to: María-Eugenia Guazzaroni, meguazzaroni@ffclrp.usp.br

Faculty of Philosophy, Sciences and Letters of Ribeirão Preto, University of São Paulo.

Av. Bandeirantes, 3.900. CEP: 14040-901, Ribeirão Preto, São Paulo, Brazil.

Supporting Information

We provided a summary of the strains used in this work along with the set of mutations identified in tolerized BJa5 and KT2440. Additionally, we showed information on the growth characterization of BJa5 and KT2440 starting strains in M9 + 2 g/L acetate + p-CA (0.016 - 0.040 - 0.080 g/L), which did not promote any inhibitory effect for both strains.

Table S1. Wild-type and tolerized strains used in this work.

Bacterium strain	Features	Reference
Pseudomonas sp. BJa5	Isolated from garden soil, Ribeirão Preto, SP (Brazil)	[1]
Pseudomonas sp. BJa5 P46 #3	Tolerized strain (replicate 3 of 6)	This work
Pseudomonas sp. BJa5 P48 #1	Tolerized strain (replicate 1 of 6)	This work
Pseudomonas sp. BJa5 P48 #4	Tolerized strain (replicate 4 of 6)	This work
Pseudomonas putida KT2440	Reference strain	[2]
Pseudomonas putida KT2440 P43 #1	Tolerized strain (replicate 1 of 6)	This work
Pseudomonas putida KT2440 P49 #3	Tolerized strain (replicate 3 of 6)	This work
Pseudomonas putida KT2440 P52 #2	Tolerized strain (replicate 2 of 6)	This work
Cytobacillus pseudoceanisediminis Chr1	Isolated from slurry waster, Nova Iguaçu, RJ (Brazil)	This work

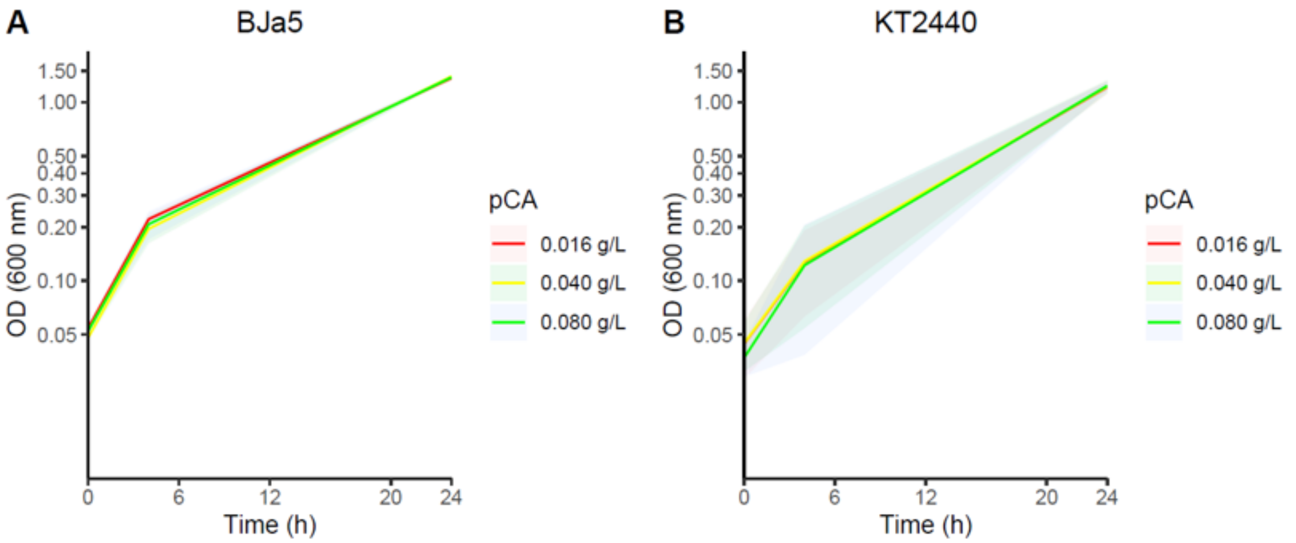

Figure S1. Growth characterization of starting strains in p-coumaric acid (*p*-CA). (A) *Pseudomonas* sp. BJa5 and (B) *P. putida* KT2440 cultured in *p*-CA gradient (0.016, 0.040, and 0.080 g/L) at 24h. The plots represent mean + SD (smooth lines) from at least three biological replicates.

Table S2. Mutated genes and intergenic regions identified in BJa5 end-strains replicates (R1-R6). The number 1 represents the presence and 0 the absence of a mutation.

Contig	Position	Alteration	R1	R2	R3	R4	R5	R6	Gene/intergenic region
2	169927	G > [T]	0	0	0	1	1	1	*acsA* (PP_4487)
1	56314	A > [G]	0	0	1	0	0	0	*acnB* (PP_2339)
1	238180	T > [C]	0	0	0	0	1	0	*tuf-A* (PP_0440)
1	238181	G > [A]	0	0	0	0	1	0	*tuf-A* (PP_0440)
12	27595	G > [A]	0	0	0	1	0	0	peptidylprolyl isomerase (PP_4313)
13	17694	C > [T]	0	0	0	1	1	1	*yhaJ* (PP_4929)
13	16072	A > [T]	0	1	0	0	0	0	*yhaJ* (PP_4929)
14	110714	G > [T]	1	0	1	0	0	0	*yhaJ* (PP_4929)
14	110716	G > [C]	0	1	1	1	0	0	hypothetical protein
15	108642	A > [C]	1	0	0	0	0	0	hypothetical protein
15	112426	G > [C]	1	0	1	0	0	0	hypothetical protein
17	121522	C > [A]	1	1	1	1	1	1	Intergenic region
19	7378	C > [G]	0	1	0	0	0	0	Intergenic region
2	169933	A > [T]	1	1	1	1	1	1	Intergenic region
2	169939	G > [C]	0	0	0	0	0	1	Intergenic region
2	169941	T > [A]	0	0	0	0	0	1	Intergenic region
20	58743	T > [C]	0	0	0	0	1	0	Intergenic region
23	36444	T > [C]	0	1	0	0	0	0	Intergenic region
23	36450	C > [G]	1	1	1	1	1	1	Intergenic region
27	57328	C > [G]	1	1	1	1	1	1	Intergenic region
27	57386	C > [A]	1	0	1	1	1	1	Intergenic region
27	57613	T > [G]	0	1	0	0	0	0	Intergenic region
27	26695	G > [C]	0	0	0	1	0	0	Intergenic region
27	26701	C > [G]	0	0	0	1	0	0	Intergenic region
3	27664	T > [G]	0	0	1	0	0	0	Intergenic region
36	8649	T > [G]	0	0	1	0	0	0	Intergenic region
5	47	A >[C]	0	0	1	0	0	0	Intergenic region
5	48	G > [C]	0	0	0	0	1	0	Intergenic region
60	409	C > [G]	0	0	0	0	1	0	Intergenic region
60	451	G > [T]	0	1	1	1	0	1	Intergenic region
60	454	G > [C]	1	0	1	0	0	1	Intergenic region
61	174	G > [A]	1	0	1	0	0	1	Intergenic region
61	531	A > [G]	1	0	1	0	0	1	Intergenic region
61	557	T > [C]	1	0	1	0	1	1	Intergenic region
64	408	C > [T]	0	0	1	0	0	1	Intergenic region
8	224189	T > [C]	0	0	1	0	0	1	Intergenic region
8	224225	A > [C]	0	0	0	0	1	0	Intergenic region
9	20508	A > [G]	0	1	0	0	1	1	Intergenic region
90	101	C > [T]	1	1	1	0	0	0	*rcsC* (PP_2664)

Table S3. Mutated genes and intergenic regions identified in KT2440 end-strains replicates (R1-R6). The number 1 represents the presence and 0 the absence of a mutation.

Contig	Position	Alteration	R1	R2	R3	R4	R5	R6	Gene/Intergenic region
20	4247	A >[G]	0	1	0	0	0	0	deaD (PP_1868)
9	64092	A > [G]	0	1	1	0	1	0	*allS* (PP_4929)
9	64137	G > [A]	1	0	0	1	0	1	*allS* (PP_4929)
3	149602	G > [C]	1	0	0	1	0	1	*allS* (PP_4929)
1	79296	A > [C]	0	0	1	0	0	0	hypothetical protein
4	215402	A > [T]	0	0	0	1	0	0	hypothetical protein
23	10529	C > [T]	1	1	1	1	1	1	hypothetical protein
23	10535	G > [C]	1	1	1	1	1	1	hypothetical protein
23	10538	C > [T]	1	1	1	1	1	1	hypothetical protein
23	10547	T > [G]	1	1	1	1	1	1	hypothetical protein
23	10556	G > [T]	1	1	1	1	1	1	hypothetical protein
23	10565	T > [C]	1	1	1	1	1	1	hypothetical protein
23	10577	T > [C]	1	1	1	1	1	1	hypothetical protein
23	10598	T > [C]	1	1	1	1	1	1	hypothetical protein
23	10643	A > [G]	1	1	1	1	1	1	hypothetical protein
23	10649	T > [C]	1	1	1	1	1	1	hypothetical protein
23	10650	T > [C]	1	1	1	1	1	1	hypothetical protein
23	10685	C > [T]	1	1	1	1	1	1	hypothetical protein
23	10778	T > [C]	1	1	1	1	1	1	*ISPpu14* (PP_4439)
23	10832	G > [A]	1	1	1	1	1	1	*ISPpu14* (PP_4439)
23	10838	G > [A]	1	1	1	1	1	1	*ISPpu14* (PP_4439)
23	10868	C > [T]	1	1	1	1	1	1	*ISPpu14* (PP_4439)
23	11072	G > [C]	1	1	1	1	1	1	*ISPpu14* (PP_4439)
23	11102	A > [G]	1	1	1	1	1	1	*ISPpu14* (PP_4439)
23	11105	C > [T]	1	1	1	1	1	1	*ISPpu14* (PP_4439)
23	11108	G > [C]	1	1	1	1	1	1	*ISPpu14* (PP_4439)
23	11138	G > [A]	1	1	1	1	1	1	*ISPpu14* (PP_4439)
23	11316	G > [A]	1	1	1	1	1	1	*ISPpu14* (PP_4439)
23	11321	C > [T]	1	1	1	1	1	1	*ISPpu14* (PP_4439)
23	11480	G > [A]	1	1	1	1	1	1	*ISPpu14* (PP_4439)
23	11546	A > [G]	1	1	1	1	1	1	*ISPpu14* (PP_4439)
23	11624	C > [T]	1	1	1	1	1	1	*ISPpu14* (PP_4439)
23	11684	C > [T]	1	1	1	1	1	1	*ISPpu14* (PP_4439)
23	11961	C > [T]	1	1	1	1	1	1	*ISPpu14* (PP_4439)
23	11987	C > [T]	1	1	1	1	1	1	*ISPpu14* (PP_4439)
23	12092	A > [C]	1	1	1	1	1	1	*ISPpu14* (PP_4439)
23	12117	T > [C]	1	1	1	1	1	1	*ISPpu14* (PP_4439)
23	12134	G > [A]	1	1	1	1	1	1	*ISPpu14* (PP_4439)
7	30701	G > [A]	0	1	1	0	1	0	*nuoC* (PP_4121)
5	251027	T > [G]	0	0	0	0	1	0	Intergenic region
6	192387	T > [G]	0	0	1	1	1	0	Intergenic region
6	192402	A > [G]	0	0	1	1	1	0	Intergenic region

14	119586	A > [G]	0	1	1	0	1	0	Intergenic region
14	119592	A > [G]	1	0	0	1	0	1	Intergenic region
23	10698	T > [A]	1	1	1	1	1	1	Intergenic region
23	10712	A > [G]	1	1	1	1	1	1	Intergenic region
23	10720	G > [A]	1	1	1	1	1	1	Intergenic region
23	10732	A > [C]	1	1	1	1	1	1	Intergenic region
23	10733	T > [C]	1	1	1	1	1	1	Intergenic region
84	593	G > [A]	0	1	1	0	1	0	Intergenic region
84	638	T > [G]	0	1	1	0	1	0	Intergenic region
84	644	C > [A]	0	1	1	0	1	0	Intergenic region
84	647	G > [A]	0	1	1	0	1	0	Intergenic region
84	654	A > [G]	0	1	1	0	1	0	Intergenic region
88	640	C > [T]	1	0	0	1	0	1	Intergenic region
25	16940	G > [A]	0	0	0	0	1	0	*mrdA-II* (PP_4807)
1	9916	A > [G]	1	0	0	1	0	1	*rhsA* (PP_3108)

References

(1) Rabiço, F.; Pedrino, M.; Narcizo, J. P.; de Andrade, A. R.; Reginatto, V.; Guazzaroni, M.-E. Synthetic Biology Toolkit for a New Species of Pseudomonas Promissory for Electricity Generation in Microbial Fuel Cells. *Microorganisms* 2023, *11* (8), 2044. https://doi.org/10.3390/microorganisms11082044.

(2) Bagdasarian, M.; Lurz, R.; Rückert, B.; Franklin, F. C. H.; Bagdasarian, M. M.; Frey, J.; Timmis, K. N. Specific-Purpose Plasmid Cloning Vectors II. Broad Host Range, High Copy Number, RSF 1010-Derived Vectors, and a Host-Vector System for Gene Cloning in Pseudomonas. *Gene* 1981, *16* (1–3), 237–247. https://doi.org/10.1016/0378-1119(81)90080-9.
